# Recognition and Matching of Clustered Mature Litchi Fruits Using Binocular Charge-Coupled Device (CCD) Color Cameras

**DOI:** 10.3390/s17112564

**Published:** 2017-11-07

**Authors:** Chenglin Wang, Yunchao Tang, Xiangjun Zou, Lufeng Luo, Xiong Chen

**Affiliations:** 1 Key Laboratory of Key Technology on Agricultural Machine and Equipment, Ministry of Education, South China Agricultural University, Guangzhou 510642, China; 2005209012@stu.scau.edu.cn (C.W.); chenxiong@stu.scau.edu.cn (X.C.); 2 School of Civil and Transportation Engineering, Guangdong University of Technology, Guangzhou 510006, China; 3 School of Mechanical and Electrical Engineering, Foshan University, Foshan 528000, China; luolufeng@tute.edu.cn

**Keywords:** litchi recognition, harvesting robot, binocular vision, stereo matching

## Abstract

Recognition and matching of litchi fruits are critical steps for litchi harvesting robots to successfully grasp litchi. However, due to the randomness of litchi growth, such as clustered growth with uncertain number of fruits and random occlusion by leaves, branches and other fruits, the recognition and matching of the fruit become a challenge. Therefore, this study firstly defined mature litchi fruit as three clustered categories. Then an approach for recognition and matching of clustered mature litchi fruit was developed based on litchi color images acquired by binocular charge-coupled device (CCD) color cameras. The approach mainly included three steps: (1) calibration of binocular color cameras and litchi image acquisition; (2) segmentation of litchi fruits using four kinds of supervised classifiers, and recognition of the pre-defined categories of clustered litchi fruit using a pixel threshold method; and (3) matching the recognized clustered fruit using a geometric center-based matching method. The experimental results showed that the proposed recognition method could be robust against the influences of varying illumination and occlusion conditions, and precisely recognize clustered litchi fruit. In the tested 432 clustered litchi fruits, the highest and lowest average recognition rates were 94.17% and 92.00% under sunny back-lighting and partial occlusion, and sunny front-lighting and non-occlusion conditions, respectively. From 50 pairs of tested images, the highest and lowest matching success rates were 97.37% and 91.96% under sunny back-lighting and non-occlusion, and sunny front-lighting and partial occlusion conditions, respectively.

## 1. Introduction

Litchi is one of the most popular fruits. The average annual yield of litchi in China was about 1.45 million tons during the last decade [1]. Therefore, it is necessary to develop a litchi-harvesting robot with the aim of automatic harvesting, which may be able to solve the problems of time-consuming and high labor-costs of litchi harvesting.

Multiple fruit harvesting robots have been developed by researchers [2,3,4,5,6,7]. In the structural unit of the robot, the vision system is the most vital fundamental module for the fruit harvesting robot to automatically pick fruit. The vision sensor is the direct source of the vision system acquiring fruit information. Different kinds of cameras integrating vision sensors have been used in the vision system; for example, thermal, hyperspectral and color cameras. After acquiring fruit images using cameras, the vision system will process and analyze the images using some methods of recognition and localization of fruit proposed by the researchers. Thus, the vision system can provide three-dimensional coordinates of the fruit in the real world to the fruit harvesting robot, hence guiding the robot to perform fruit harvesting.

In order to overcome the influences caused by the natural environment such as varying illumination, random occlusion and color similarity, many studies have been reported by resreachers, which analyzed fruit images acquired by using cameras integrating vision sensors for the recognition and localization of fruit. However, there has not been an ideal method yet. Bulanon et al. [8] used a thermal camera to acquire thermal images of citrus to identify and locate the harvesting target. Wachs et al. [9] adopted a computer vision system compried of an infra-red camera and a color camera for apple detection. The Infra-red camera provided clues regarding the physical structures and locations of the apples based on their temperatures. The color camera provided evidence of circular shape. In [10], a near-infrared (NIR) camera was used to capture citrus images at three optical bands (1064, 1150 and 1572 nm). Thus, green citrus could be identified using the index calculation that was done based on images at three different wavelengths. Safren et al. [11] analyzed hyperspectral data in visible and NIR ranges by using a proposed algorithm for apple recognition. The recognition accuracy of the apple fruit achieved 88.1%. Although the above detection methods can achieve higher correct fruit detection rates by using advanced vision sensors and fusion of multiple image data, the feasibility of analysis of hyperspectral and thermal imaging for developing a fruit-harvesting robot is limited by time-consuming data acquisition, expensive devices and complicated image analysis processes [11].

Direct analysis of fruit color images acquired using a much less expensive red-green-blue (RGB) color camera may be a more feasible method for fruit detection and fruit harvesting robot development. Numerous articles focused on RGB color image analysis-based detection algorithm of fruit. Rajendra et al. [12] proposed a machine vision algorithm for robots to harvest strawberries in tabletop culture greenhouses. They used three charge-coupled device (CCD) cameras to capture the strawberry image, in which the right and left cameras were used to calculate the stereo depth of the object from the camera. The center camera was used to detect the target strawberry, correct the position and detect the peduncle and the angle of peduncle. Therefore, the strawberry harvesting robots developed, based on their mentioned method, could harvest 76.55% of strawberries in a real greenhouse. Bulanon et al. [13] used an RGB color camera combining an artificial lighting to acquire apple images. The R color component difference between the objects was proposed to detect apples. The accuracy achieved 88.0%, which was obtained under controlled lighting conditions, whereas the accuracy was not reported under uncontrolled lighting conditions. Arefi et al. [14] extracted ripe tomatoes from tomato color images using the method of combination of RGB, hue, saturation and intensity (HSI), luminance, in-phase and quadrature-phase (YIQ) spaces. The extracted ripe tomatoes were located using morphological features. The total accuracy from 110 color images was 96.36%. The accuracy was obtained under greenhouse light conditions. In [15], pomegranate fruits on trees were detected based on analysis of pomegranate color images acquired under grove conditions. However, the author recognized that although the proposed method combining color and shape of the pomegranate was robust, the accuracy of detection rate was affected by lighting and occlusion. In [16], in order to reduce the impact of mango fruit occlusion and lighting conditions, a method for mango recognition was reported. The method was based on color analysis in the RGB and luminance, blue chrominance and red chrominance (YCbCr) color spaces, and texture segmentation using adjacent pixel variability. However, fruit recognition results would be ideal under consistent lighting conditions in the mango grove. In [17], kiwifruits were recognized at night time by extracting R-G color components. The success rate of the recognition algorithm was 88.3% under artificial lighting conditions. Luo et al. [18] used an H-I color component-based fuzzy clustering method to segment the grape color image acquired in the vineyard. The accuracy was 90.33% and most of the missed fruit happened under occlusion conditions. 

It is clear from the above fruit detection approaches that fruit detection based on analyzing RGB color images is challenged by varying illumination and occlusion conditions. In the case of litchi fruit detection using color images, Xiong et al. [19] proposed monocular vision-based mature litchi fruit recognition. The algorithm firstly converted RGB color space of original litchi color images into YCbCr color space. Then the fuzzy C-means clustering algorithm was used to separate the fruit from the Cr component image. The recognition rate under different natural conditions reached 95.5%, however, the method only recognized fruits based on color analysis, which had no potential for the recognition of the partially occluded and clustered fruits. Guo et al. [20] explored litchi segmentation methods based on different color spaces. The results indicated that when the threshold value of Cr was between 0.50 and 0.54, litchi fruits and their main fruit-bearing branches could be segregated from the cluster of litchi. The recognition ratios of the fruits and the main fruit-bearing branches were 91.67% and 95.00%, respectively. The author recognized varying illumination under the natural environment was the reason of missed fruits. Wang et al. [21] firstly applied a wavelet transform to normalize illumination of litchi image surface for reducing the influence of varying illumination. Then mature litchi fruits were separated from leaves, branches and background using color-based *K*-means clustering. Although the highest average recognition rates for non-occluded and partially occluded litchi were 98.8% and 97.5%, respectively, the recognition method was based on red color component extraction, which did not take into account various characteristics of litchi such as texture features and clustered growth.

From the above, we see that current color image analysis-based studies for mature litchi detection in the natural environment have focused on color-based segmentation, whose main aims were to identify the mature litchi targets for the litchi harvesting robot [19,20,21]. However, it was difficult to identify a mature litchi target only by relying on one kind of color space classification in the complicated natural environment. In recent years, machine learning technology has become popular among researchers. More and more visual learning approaches have been used for fruit detection in the natural environment [22]. Chinchuluun et al. [23] developed a citrus detection approach based on Bayesian classifier by selecting S and I components of hue, saturation and value (HSV) color space and YIQ color space, respectively. In [24], a method to detect fruits was proposed, in which apples, bananas, lemons and strawberries were identified by extracting fruit color, shape, area and perimeter features, and classifying them by the k-NearestNeighbor (KNN) classifier. In [25], an apple detection mechanism was proposed. To improve the detection accuracy, the multi-layer neural network trained with the pixel value of fruit and background in luminosity, the range from magenta to green and the range from yellow to blue (La*b*), HSV and RGB color spaces were used to identify candidate targets. In [26], a citrus detection approach was designed by establishing a multi-class support vector machine (SVM) classifier, in which the radial basis function (RBF) kernel function was used to classify citrus, leaves and branches based on RGB color features. 

In the case of fruit matching using color images, Jian et al. [27] proposed a method based on binocular stereo vision technology for apple matching. After apple recognition using the color index R − G > 0 and (R − G)/(G − B) > 1, Si et al. [28] used a matching algorithm based on area and epipolar geometry to match the apples. In [21], Wang et al. proposed a label template-based matching algorithm to match litchis. However, the above methods are for single fruit matching. It is difficult to match a single target under random occlusion conditions in the natural environment.

Inspired by fruit recognition based on the color image segmentation methods, the machine learning methods and the fruit matching methods, this paper mainly focused on the combination of the three methods to find a robust method for recognition and matching of clustered mature litchi fruits. In the litchi orchard, varying illumination, random litchi growth such as clustered growth with the uncertain number of the fruit and random occlusion by leaves, branches and other fruits make the robustness of unsupervised machine learning methods difficult. Therefore, in order to eliminate the influences caused by complicated environments and improve the detection rate of litchi fruits in the orchard, a method of recognition and matching of clustered mature litchi fruits was developed based on litchi color images acquired by binocular CCD color cameras. Firstly, after calibration of binocular color cameras, litchi image acquisition was carried out under litchi orchard conditions. Due to the randomness of litchi growth, such as clustered growth with the uncertain number of fruits, the mature litchi fruit clustering patterns were manually defined as three categories by analyzing litchi growth. They were single litchi fruit (category A), two clustered litchi fruits (category B) and multiple clustered litchi fruits (category C). They were also the most common cases of clustered litchi fruits. By defining the three categories, recognition and matching can be easily implemented. Secondly, litchi fruits and non-fruits were manually cropped from litchi images. Four different kinds of classifiers were trained by using the features of litchi fruits and non-fruits. After combining the classification results of the four classifiers, a pixel threshold method was presented for recognizing the pre-defined categories of clustered litchi fruit. Finally, a geometric center-based matching method was proposed for matching the recognized clustered litchi fruit.

## 2. Materials and Methods

### 2.1. Calibration of Cameras and Image Acquisition

**Figure 1:**
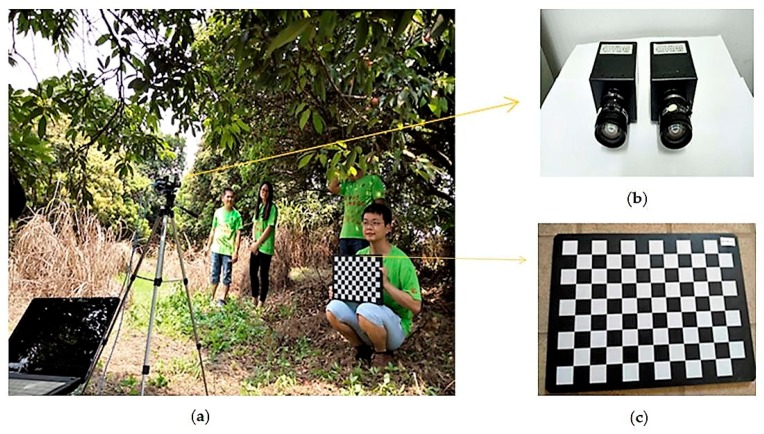
The proposed system figure: (**a**) Calibration of cameras; (**b**) Two CCD color cameras; (**c**) A calibration board.

As shown in Figure 1a, the proposed system of recognition and matching of clustered mature litchi fruits consisted of a personal computer (PC), two CCD color cameras, a tripod, a calibration board, two cables and a software system. The cameras (model MV-VD120SC, supplied by Microvision company in Xi’an, China), as shown in Figure 1b, which integrated CCD vision sensors, had a digital video output of 1280 by 960 effective pixels, and were parallel mounted on the tripod. The distance between centers of the two camera lenses was fixed at 200 mm in this study. The focal length of the cameras was selected as 6 mm. The software system was developed and ran on the PC with 4 GB RAM, an Intel Core i5-2500 CPU and a Windows 7 operating system. The two cables have two USB2.0 interfaces, which can be used for the two cameras connecting to the two interfaces of PC, respectively. The acquired litchi color images were processed and analyzed by the software system, which was composed by cameras calibration module programmed in OpenCV 3.0 (supplied by Intel Corporation at Santa Clara, CA, USA), recognition module of clustered litchi fruit and matching module of clustered litchi fruit programmed in Matlab 8.3 (supplied by MathWorks Corporation at Nedik, MA, USA).

In order to determine the parameters of the cameras and obtain real-world coordinates, camera calibration is a necessary process. In a binocular vision system, the aim of the binocular calibration is to calculate the relative positional relationship between the two cameras, that is, to obtain the rotation matrix R and the translation vector T between the two cameras [29]. For each camera, the representation of the point of the real-world coordinate system in the image coordinate system is shown in Equation (1),

(1) $$c\left[\begin{array}{c} \mu \\ \gamma \\ 1 \end{array}\right]=\left[\begin{array}{ccc} a_{x} & 0 & \mu_{0}\\ 0 & a_{y} & \gamma_{0}\\ 0 & 0 & 1 \end{array}\;\begin{array}{c} 0\\ 0\\ 0 \end{array}\right]\left[\begin{array}{cc} R_{r/l} & T_{r/l}\\ 0^{T} & 1 \end{array}\right]\left[\begin{array}{c} x_{w}\\ y_{w}\\ \begin{array}{c} z_{w}\\ 1 \end{array} \end{array}\right]=M_{1}M_{2}X_{w}$$

where c is a constant, (μ,γ) is the coordinates of point in the image coordinate system, $\left(x_{w},y_{w},z_{w}\right)$ is the point in the real world coordinate system and $X_{w}={\left(x_{w},y_{w},z_{w},1\right)}^{T}$. $a_{x}$, $a_{y}$, $\mu_{0}$ and $\gamma_{0}$ are the internal parameters of each camera, which are represented by the internal parameter matrix $M_{1}$. $R_{r/l}$ is a 3 × 3 orthogonal matrix, $0^{T}=\left(0,0,0\right)$ and $T_{r/l}$ is a 3 × 1 translation vector, which are represented by the external parameter matrix $M_{2}$. The camera calibration requires a precise positioning of the lattice template. As shown in Figure 1c, a calibration board of 11 × 8 corner points (model AFT-MCT-0V430, supplied by Microvision Company in Xi’an, China) was used in this study for camera calibration. The two cameras were fixed, and the distances between the cameras and the calibration board were adjusted for making sure that each corner point of the calibration board is in the field of view of each camera. Then, the calibration board pose relative to the cameras was changed. Each camera captured 15 effective images of the calibration board, which could be obtained by changing 15 poses relative to the cameras at the same position, and where all of the corner points had to be extracted. The origin of the real-world coordinate system was selected as the upper left corner of the calibration board. The x-axis was horizontally right and the y-axis was vertically down. Thus, the position of the corner points of the calibration board in the real-world coordinate system and the pixel coordinates of the corner points in all the images could be obtained. The internal and external parameters of each camera could be calculated by using Equation (1). Thus, R and T could be calculated by using Equation (2).

(2) $$R=R_{r}^{-T}R_{l}^{T},\;T=T_{r}-R^{-T}T_{l}$$

After calibrating the cameras, litchi color images from the orchard under sunny and cloudy daylight conditions were obtained.

### 2.2. Algorithm Description

**Figure 2:**
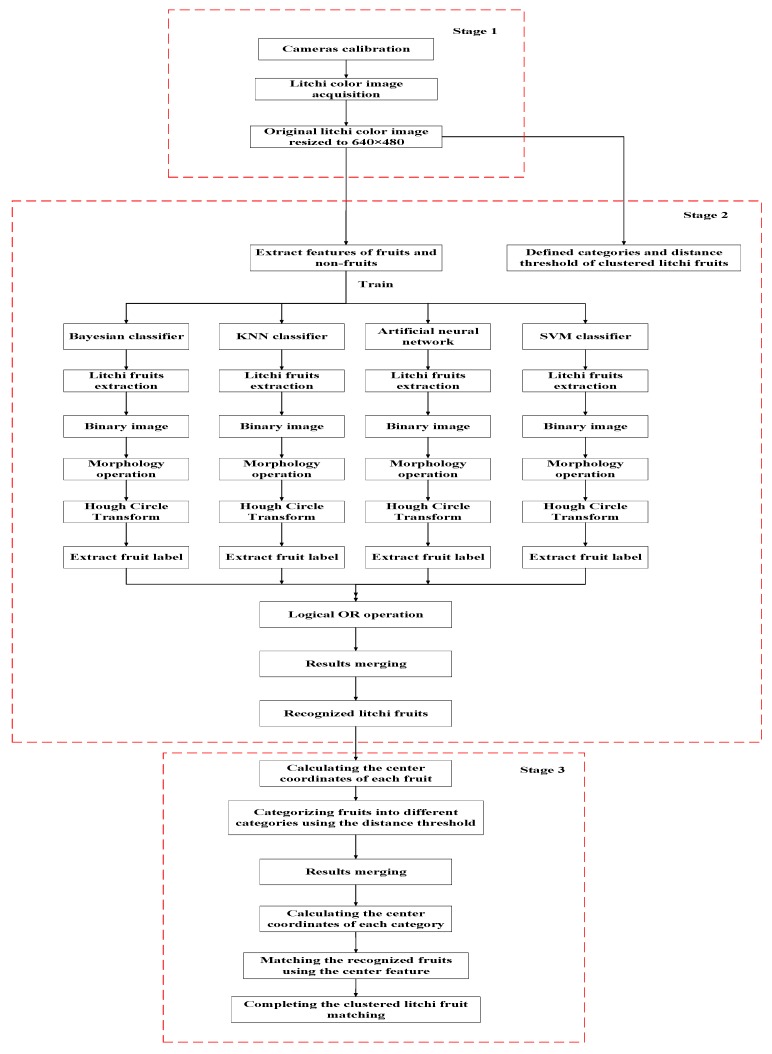
Flow chart of the proposed algorithm.

As shown in Figure 2, the proposed algorithm included three stages: calibration of cameras and image acquisition, recognition of clustered litchi fruit and matching of clustered litchi fruit. In stage 1, after calibration of cameras, the acquired original litchi color image was 1280 × 960 pixels. For convenient image processing, the litchi image was firstly resized into 640 × 480 pixels. In stage 2, by analyzing the litchi image, the categories of clustered litchi fruits were pre-defined, which were divided into three categories based on the distance between the centers of any two litchi fruits. In parallel, four effective color components and six primary visual features of fruits and non-fruits were extracted. Then, the four different classifiers, Bayesian classifier, KNN classifier, artificial neural network and SVM classifier, were trained by the features, respectively. Therefore, litchi fruit extraction of the same image can be implemented using the four trained classifiers, respectively. Then, each extracted result image was transformed into a binary image, where morphology operations were used to remove noise and fill the hole. After finishing litchi fruit region detection using circle hough transform (CHT), each litchi fruit label was extracted. Lastly, litchi fruit recognition was completed by merging the four label extraction results of the same image based on logical OR operation. In stage 3, label center coordinates of each recognized fruit were firstly calculated. Then, the fruits were assigned into the pre-defined categories using the distance threshold between the two label centers. After merging the category result of each fruit, the label center coordinates of each category were calculated. Finally, the recognized clustered litchi fruits were matched using a matching method based on clustered fruit label. All the details will be shown in the following sections.

### 2.3. Category Definition of Clustered Litchi Fruit

**Figure 3:**
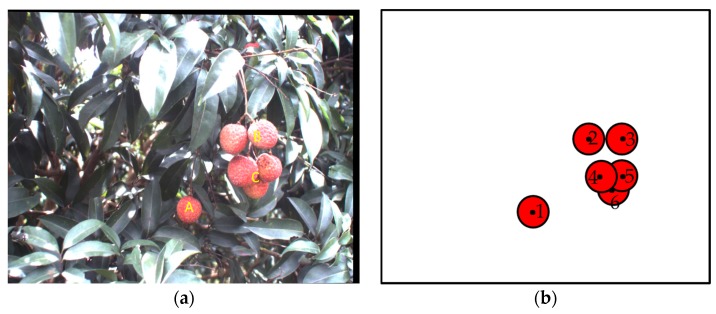
The figure of category definition of clustered litchi fruit: (**a**) A typical litchi canopy image, A is single litchi fruit, B are two clustered litchi fruits, C are multiple clustered litchi fruits; (**b**) An explanation image of clustered litchi fruit category, 1 belongs to single litchi fruit, 2 and 3 belong to two clustered litchi fruits, 4, 5 and 6 belong to multiple clustered litchi fruits.

Figure 3a is a typical litchi canopy image acquired under the natural environment, which is also the left image in the stereo images, and has been implemented the image alignment processing. In the image, it can be seen that the growth of litchi fruits has randomness. Regions A, B and C, which correspond to fruit 1, fruits 2, 3 and fruits 4, 5 and 6, were shown in Figure 3a,b, respectively. After resizing the original litchi color image into 640 × 480 pixels, the average diameter of the single litchi fruit is about 40 pixels confirmed in the training set. Therefore, categories of clustered litchi fruit can be defined as the most common three cases as follows:

- Single litchi fruit (category A): If the Euclidean distance between the geometric center of one litchi fruit and the geometric center of any other litchi fruit is greater than the average diameter of the single litchi fruit (i.e., 40 pixels), the litchi fruit will be defined as the single litchi fruit like A region;
- Two clustered litchi fruits (category B): If the Euclidean distance between the geometric centers of only any two litchi fruits is smaller than the average diameter of the single litchi fruit (i.e., 40 pixels), the litchi fruits will be defined as the two clustered litchi fruits like B region;
- Multiple clustered litchi fruits (category C): If the Euclidean distance between the geometric centers of more than two litchi fruits is smaller than the average diameter of the single litchi fruit (i.e., 40 pixels), the litchi fruits will be defined as the multiple clustered litchi fruits like C region.

### 2.4. Recognition Algorithm of Category of Clustered Litchi Fruit

#### 2.4.1. Analysis and Extraction of Features of Mature Litchi Fruit and Non-Fruit

**Figure 4:**
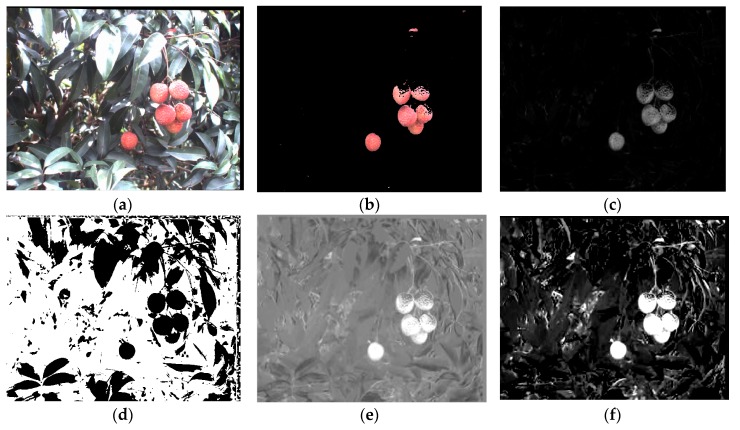
Analysis images of effective color components. (**a**) Original litchi color image; (**b**) Mature litchi fruit extraction based on only red color component; (**c**) R-B component image; (**d**) I component image; (**e**) Cb component image; and (**f**) b* component image.

Shape, color, and texture-based analyses are usually used to detect fruits in a color image [28]. As shown in Figure 4a, although the litchi shape is basically conical in space, the shape is irregular, round and sometimes approximates to a triangle in two-dimensions. It is difficult to apply a simple algorithm for detecting litchi fruits. Therefore, the shape-based analysis should not be used for detecting litchi fruits [21]. The color of the mature litchi fruit is red, which is different from the color of other objects on a litchi tree. However, no ideal results were obtained from extracting litchi fruits using only the red color component to segment the image under RGB color space, which made some light spot on litchi fruit surfaces as shown in Figure 4b. It is necessary to find more effective color components that can separate the litchi fruits from the background and keep the fruits intact. Meanwhile, considering the different textures between the fruit and the background, texture-based analysis can be a potential method for separating the fruits from background. Thus, color-based analysis and texture-based analysis were applied for the extraction of the mature litchi fruits in this study.

The litchi color images were transformed from RGB color space to HSI color space, YCbCr color space and L*a*b color space. By extracting color components of each color space, the color difference between the litchi fruits and background (e.g., the leaves, branches, sky and so on) was contrasted by using the visual evaluating methods as shown in Figure 4. From the figure, it can be seen that the R-B, I, Cb and b* components were the more effective color components. Tamura et al. [30] proposed six basic texture features based on human subjective psychological measures as criteria, which are coarseness, contrast, directionality, line-likeness, regularity and roughness, and are all widely used in image segmentation. Thus, the six texture features were used to represent the texture features of litchi fruits. By substituting manually cropped litchi fruit images into corresponding equations stated in [30], the six texture features can be generated and the values of the six features can be calculated. Therefore, the effective color components and texture features were selected to construct the classifiers for extracting mature litchi fruits from the litchi color image.

#### 2.4.2. Construction and Training of Four Kinds of Classifiers

**Figure 5:**
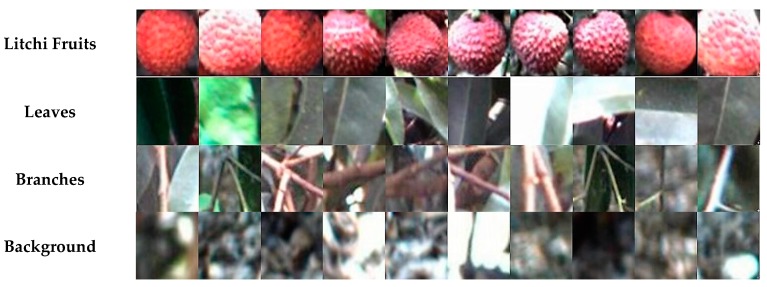
Examples of the dataset images. The top row listed the images of mature litchi fruits captured under the natural condition, and the lower rows were leaves, branches and background.

Naive Bayes classifier, KNN classifier, artificial neural network and SVM classifier are supervised learning techniques, which are most commonly used for automatic classification [31]. Therefore, these four classifiers were used to extract mature litchi fruits in this study. In order to train these classifiers, 20 images were randomly selected from the 70 images captured under the natural condition, from which 150 mature litchi fruit samples and background samples were manually cropped as the training dataset. For convenient image processing, these samples were uniformly processed into 40 × 40 pixels using Matlab, which was also the average size of the single litchi fruit as shown in Figure 5.

The construction and training of these classifiers using the features of litchi fruit and non-fruit are described below. We set feature vector $X=\left(x_{1},\cdots ,x_{j},\cdots ,x_{10}\right)$ as the training sample, in which j=10 and $x_{j}$ represented the values of four effective color components and six primary visual features of fruit and non-fruit stated in the previous section, respectively. $C=\left(c_{1},c_{2}\right)$ represented classes, in which $c_{1}$ and $c_{2}$ were fruit class and non-fruit class, respectively.

- Naive Bayes classifier is a simple probabilistic classifier based on applying Bayes theorem with strong independence assumptions, which can predict the probability that a given sample belongs to a certain category. The training and classification processes using naive Bayes classifier for mature litchi fruit recognition are as follows. We used a 40 × 40 sub-window to slide on the image for searching the fruit, and use the posterior probability shown as Equation (3) [32] to judge whether the sub-window the fruit region is.
  (3) $$P\left(C=c_{i}|X\right)=\frac{P(X|C=c_{i})P\left(C=c_{i}\right)}{P\left(X\right)}$$
  In which $P(X|C=c_{i})$ and $P\left(C=c_{i}\right)$ could be obtained by doing statistics of training sample and calculating the probability of $c_{i}$ in the training sample, respectively. And P(X) can be obtained by calculating the total probability. If $P\left(C=c_{1}|X\right)\;>\;P\left(C=c_{2}|X\right)$, the sub-window is the fruit region, or it is the non-fruit region.
- The KNN is used to test the degree of similarity between documents and k training data, and to store a certain amount of classification data, thereby determining the category of test documents. This method is an instant-based learning algorithm that categorizes objects based on closest feature space in the training set. The training and classification processes using the KNN classifier for mature litchi fruit recognition are as follows. We set $Y=\left(y_{1},\cdots ,y_{j},\cdots ,y_{10}\right)$ as the testing sample, in which j=10 and $y_{j}$ represented the value of four effective color components and six primary visual features of the testing sample, respectively. Use the cosine similarity shown as Equation (4) [33] to measure the similarity between Y and X.
  (4) $$sim\left(X,Y\right)=\frac{\sum_{i}^{10}x_{i}\cdot y_{i}}{\sqrt{\sum_{i}^{10}x_{i}^{2}}\cdot \sqrt{\sum_{i}^{10}y_{i}^{2}}}$$
  Calculate all of the distances between Y and all X using the Equation (4), and sorting the distances in ascending. Then, take the first k samples and calculate both of the percent of $c_{1}$ and $c_{2}$ in the first k samples. Lastly, classify Y into the corresponding class based on the larger percent between $c_{1}$ and $c_{2}$.
- Artificial neural network is a description of the first order characteristics of the human brain system, which is a mathematical model consisting of many simple parallel processing units. In this study, we selected BP neural network as the artificial neural network classifier. The training and classification processes by using the BP neural network for mature litchi fruit recognition are as follows. BP neural network with three layers was applied and 3 × 3 neighbor pixels with feature X were selected as the input neuron. The function of the neuron for inputting and outputting was f(x)=11+e−x. The neuron number J of intermediate layer was determined by using Equation (5) [34].
  (5) $$J=\sqrt{m+n}+a$$
  In which m and n indicated the number of inputting neuron and outputting neuron, respectively. And a was an integer between 1 and 10. In this study, the number of outputting neuron was determined as 1. Its value has 2, 1 and 0, respectively, indicating whether the tested point belonged to mature litchi fruit or not. Therefore, m=9 and n=1. J was taken any integer between 5 and 14. We set J as 7 to segment mature litchi fruit in this study.
- SVM is one of the discriminative classification methods which are commonly recognized to be more accurate. The SVM classification method is based on the structural risk minimization principle from computational learning theory. The training and classification processes using the SVM for mature litchi fruit recognition are as follows. We used vectors $\left(x_{j},c_{j}\right)$ as training feature vectors, in which $x_{j}$ represented the value of the effective color and texture components of fruit and non-fruit, and $c_{j}$ represented the class labels satisfying the following equation [35].
  (6) $$c_{j}=\{\begin{array}{c} 1,x_{j}\in A\\ -1,x_{j}\in B \end{array}$$
  In which A and B indicated fruit region and non-fruit region, respectively. Linear optimal discriminant function is shown in Equation (7).
  (7) $$f\left(x\right)=sgn\left(w^{*}x+b^{*}\right)$$
  where $w^{*}$ and $b^{*}$ were the global optimal solutions obtained by using the linear discriminant function shown as Equation (8) to obtain the minimum solution of Equation (9).
  (8) $$y_{j}\left(w^{T}x_{j}+b\right)-1\geq 0$$
  (9) $$\varphi \left(w\right)=\frac{1}{2}{∥w∥}^{2}$$
  The testing feature vectors $x_{j}$ were substituted into Equation (7) to solve the f(x). If f(x)=1, $x_{j}$ belonged to A, that was the fruit region. Or $x_{j}$ belonged to B, that was the non-fruit region.

#### 2.4.3. Litchi Fruit Recognition Based on the Combination of Four Kinds of Classifiers

**Figure 6:**
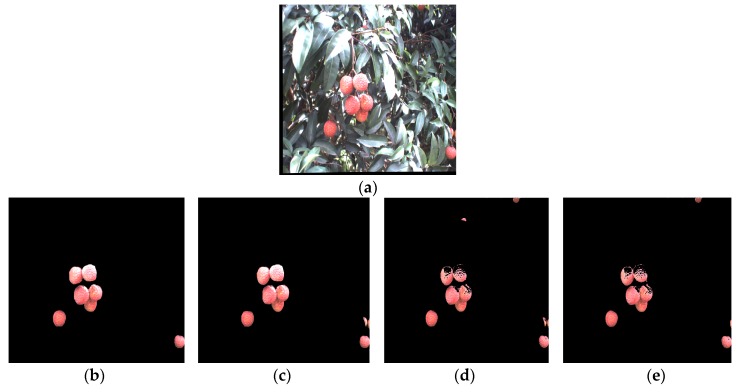
Mature litchi fruits extraction. (**a**) Original litchi color image; (**b**) Mature litchi fruit extraction based on Naive Bayes classifier; (**c**) Mature litchi fruit extraction based on KNN; (**d**) Mature litchi fruit extraction based on Back Propagation (BP) neural network; (**e**) Mature litchi fruit extraction based on support vector machine.

**Figure 7:**
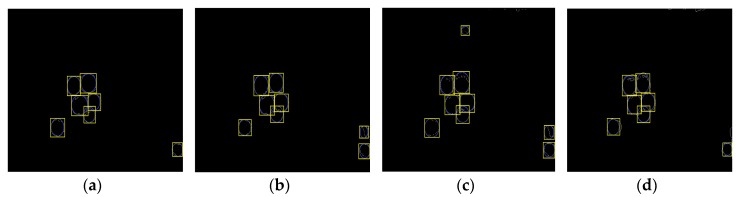
The label extraction of mature litchi fruits. (**a**) The fruit label extraction based on the segmentation result of naive Bayes classifier; (**b**) The fruit label extraction based on the segmentation result of KNN; (**c**) The fruit label extraction based on the segmentation result of Back Propagation (BP) neural network; (**d**) The fruit label extraction based on the segmentation result of support vector machine.

**Figure 8:**
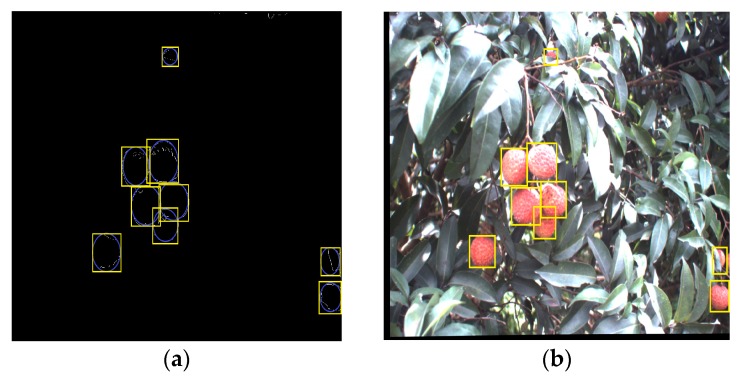
Final recognition of mature litchi fruits. (**a**) Final label extraction combining the label extractions based on the four kinds of classifiers; (**b**) Final recognition of mature litchi fruit based on the label.

After segmenting the original litchi color image (Figure 6a) using the four different kinds of classifiers described in the previous section, the extraction results of mature litchi fruits could be obtained as shown in Figure 6b–e. The images, including the extracted fruits, were converted into binary images, in which the mathematical morphology dilation operation was applied for filling the missing small areas on the fruit surfaces. ‘Disk’ was selected as the structuring element in dilation operation, and its value was 10. Then, the canny edge detection was implemented for extracting the fruit region edges. In the training set, the minimum and maximum radii of the mature litchi fruits were found to be approximately 30 and 50 pixels, respectively. Using these radius ranges, CHT could be performed on the binary level images of the edge extraction results using Matlab. ‘Imfindcircles’ was selected as the CHT function, and the parameter ‘sensitivity’ was set to 0.97. We selected the Euclidean distance of any two circle centers as the threshold to judge whether any two circles were the same one. The value of the threshold was set at 15 pixels. If the Euclidean distance between any two circle centers was smaller than 15, the two circle would be determined to be the same one. Lastly, the minimum circumscribed rectangle of each detected circle was drawn for representing the label of each mature litchi fruit as shown in Figure 7a–d. Using the ‘OR’ operation, the final labels of the mature litchi fruits could be obtained (Figure 8a) by combining the results of the four classifiers, thus, the original litchi color image is overlapped as shown in Figure 8b.

#### 2.4.4. Categories Recognition of Clustered Litchi Fruit Based on the Pixel Threshold Method

Based on the label extraction of mature litchi fruits in the previous section, the coordinates of the center of each circle and four vertices of the label were stored as $\left(x_{i},y_{i}\right)$ and $\left(x_{i}^{j},y_{i}^{j}\right)$, respectively, in which *i* indicated the number of circle centers and the value of *j* ranged from 1–4, which indicated the number of the four vertices of the corresponding minimum circumscribed rectangle. Therefore, the Euclidean distance between any two circle centers was calculated. Compared to the preset threshold (40 pixels) stated in Section 2.3, the mature litchi fruit represented by both of the circle and the label would be categorized as follows:

- If the Euclidean distance between one circle center and the center of any circle was greater than the threshold, the litchi fruit represented by the circle would be categorized as a single litchi fruit. The coordinates of its circle center and four vertices of its label would not change.
- If the Euclidean distance between the centers of only any two circles was smaller than the threshold, the litchi fruits represented by the two circles would be categorized as two clustered litchi fruits. The coordinates of their circle centers were deleted. The labels were combined into a large label, whose four vertices were the minimum and maximum abscissas and the minimum and maximum ordinates of the two labels.
- If the Euclidean distance between the centers of more than two circles was smaller than 40 pixels, the litchi fruits represented by the circles would be categorized as multiple clustered litchi fruits. The coordinates of their circle centers were deleted. The labels were combined into a large label, whose four vertices were the minimum and maximum abscissas and the minimum and maximum ordinates of all the labels.

**Figure 9:**
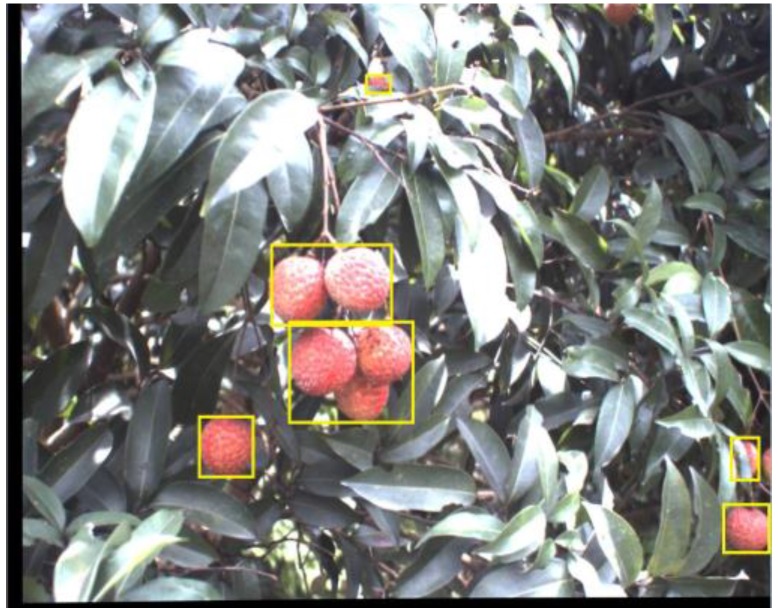
Image of categories recognition of clustered litchi fruit.

Using the above process, the categories recognition of clustered litchi fruit based on the pixel threshold were completed as shown in Figure 9.

### 2.5. Matching Method for the Recognized Categories of Clustered Litchi Fruit

#### 2.5.1. Geometric Center-Based Clustered Litchi Fruit Matching

Matching based on binocular stereo vision was the process that searched for the corresponding point of left image in the same image scene in the right image. This search process could be seen as a template matching process. CHT-based matching method has been applied in fruit matching [26,27]. However, it is difficult to select one point to represent the single fruit due to the random occlusion, which can be called as the feature point. As discussed in the previous section, the label of the clustered litchi fruit has been extracted. Therefore, the feature point could be selected as the diagonal intersection point of the label. The label of the left image was considered as the template, and it was traversed into the right image along the epipolar line to find the most similar window; the diagonal intersection point of the found window was the matching point of the clustered litchi feature point of the left image. The similarity measure method was selected with the normalized cross-correlation (NCC) based on the gray value matching shown as Equation (10) [21], which was invariant to all linear illumination changes, and hence was suitable for natural environments.

(10) $$NCC\left(d\right)=\frac{\sum_{i=1}^{M}\sum_{j=1}^{N}\left[I_{1}\left(u+i,v+j\right)-{\bar{I}}_{1}\left(u,v\right)\right]\left[I_{2}\left(u+i-d,v+j\right)-{\bar{I}}_{2}\left(u-d,v\right)\right]}{\sqrt{\sum_{i=1}^{M}\sum_{j=1}^{N}{\left[I_{1}\left(u+i,v+j\right)-{\bar{I}}_{1}\left(u,v\right)\right]}^{2}}\sqrt{\sum_{i=1}^{M}\sum_{j=1}^{N}{\left[I_{2}\left(u+i-d,v+j\right)-{\bar{I}}_{2}\left(u-d,v\right)\right]}^{2}}}$$

where (u,v) was the diagonal intersection point coordinate of label template of clustered litchi fruit in left image, the size of the template was M×N. $I_{1}\left(u+i,v+j\right)$ was the grey value of point (u+i,v+j), and ${\bar{I}}_{1}\left(u,v\right)$ was the average value of grey values of label template. The coordinate (u+i−d,v+j) was the translation result of the coordinate (u+i,v+j) in right image, and similarly, $I_{2}\left(u+i-d,v+j\right)$ was the grey value of point (u+i−d,v+j). ${\bar{I}}_{2}\left(u-d,v\right)$ was the average value of grey values of a M×N size window that its diagonal intersection point coordinates was (u−d,v). d could be obtained by solving Equation (10), which made NCC(d) reach the maximum value. Thus, the disparity d was calculated and the matching point coordinate of (u,v) was obtained.

#### 2.5.2. Implementation of Clustered Litchi Fruit Matching Algorithm

A sub-window was the minimum circumscribed rectangle of clustered litchi fruit contour, and its sides were parallel to the x and y axes of the image. Therefore, the sub-window as the label of the clustered litchi fruit could be easy to match due to its regular graph. The label of the fruit in the left image was considered as a template, which was to be traversed in the right image for searching the matching object. When the match of two labels reached a preset threshold, the match ended. In this paper, when 0.6 was chosen as the threshold, the matching achieved a satisfactory result. Some matching constraints needed to be noted for obtaining the correct matching. Based on the ordering constraint, if a litchi fruit was on the left side of another in the left image, it should also keep the same order in the right image. If there were no litchi fruits on the left side of the litchi in the right image, the corresponding position of the litchi could not be determined. Under this condition, this kind of litchi should be ignored. If some portion of a clustered litchi fruit needed to be matched in left image, and the whole cluster or some part of the cluster was in the right image, the preset threshold would be used to constrain the correct matching. The epipolar constraint stated that for the mapping point on an image, its match point must fall on another image of epipolar online. In a binocular stereo vision system, this epipolar constraint might mean that the matching points were in the same horizontal line, and *Y* values of coordinate of the matching points in the two images were equal. Therefore, matching in the different rows should be discarded. Furthermore, by triangulation, if the baseline is shorter, both the position of litchi in the left and right images will be more consistent, which makes the matching of features easier. The shorter baseline will also make distance measurement errors larger. We fixed the baseline at 200 mm in this study.

**Figure 10:**
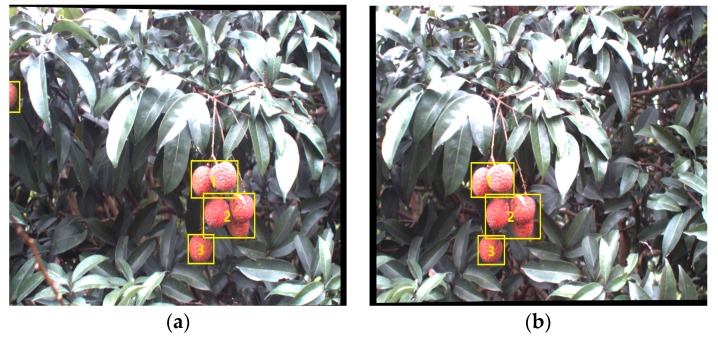
Clustered litchi fruits matching based on the proposed method. (**a**) left image; (**b**) right image.

Using the above sections, clustered litchi fruit matching result was shown in Figure 10. And 50 pairs of images were used in the matching experiment. A total of 432 pairs of clustered litchis in both the left and right images were tested in the matching experiment, in which there were 324 pairs of partially occluded litchis and 108 pairs of non-occluded litchis, respectively.

## 3. Results

### 3.1. Recognition of Clustered Litchi Fruit under Natural Environment Conditions

**Figure 11:**
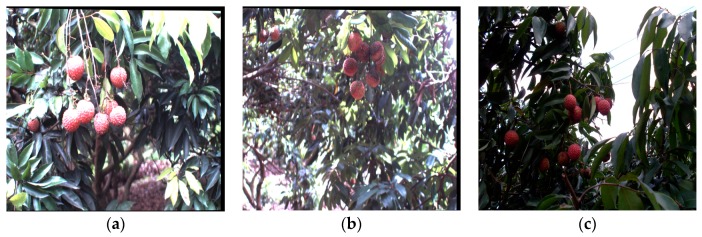
Original litchi color images under different illumination conditions. (**a**) Original litchi color image under front-lighting conditions; (**b**) Original litchi color image under back-lighting conditions; (**c**) Original litchi color image under cloudy day conditions.

**Figure 12:**
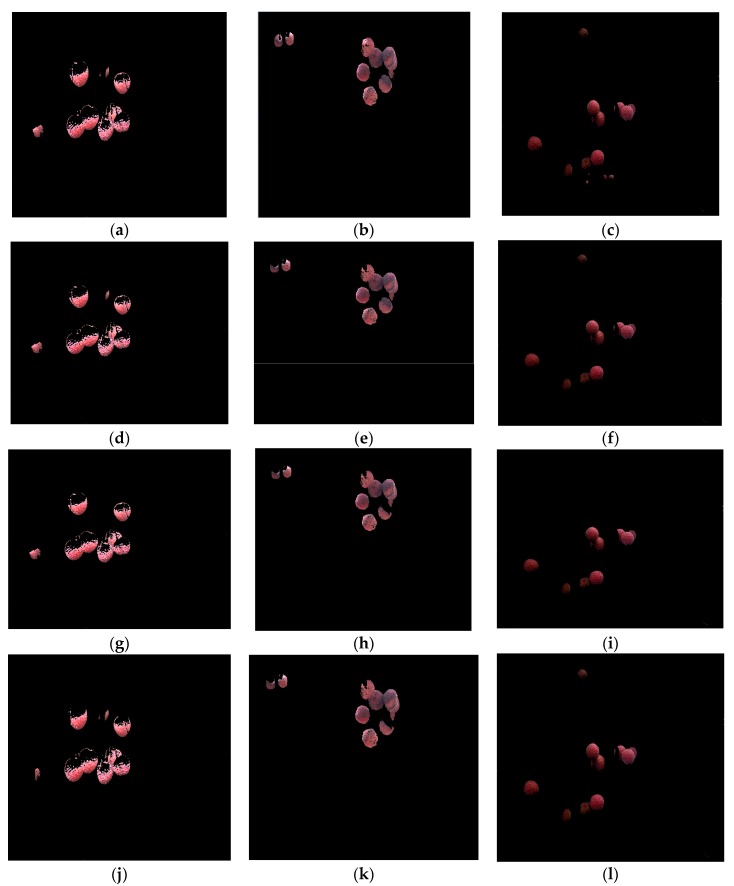
Litchi fruit extraction result by using different classifiers under different illumination conditions. (**a**–**c**) Litchi fruit extraction result by using the naive Bayes classifier under different illumination conditions; (**d**–**f**) Litchi fruit extraction result by using the KNN classifier under different illumination conditions; (**g**–**i**) Litchi fruit extraction result by using the BP neural network classifier under different illumination conditions; (**j**–**l**) Litchi fruit extraction result by using the SVM classifier under different illumination conditions.

**Figure 13:**
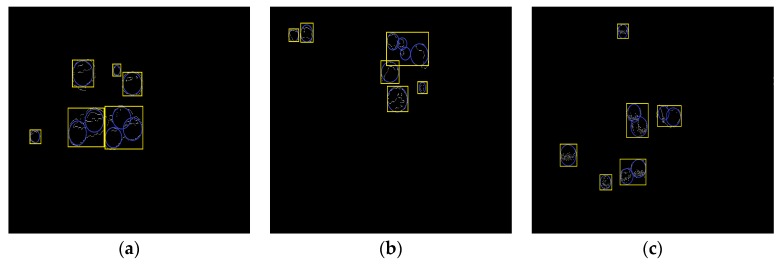
The extraction results of mature litchi fruits based on combining the four different classifiers under different illumination conditions. (**a**) The extraction results obtained under front-lighting conditions; (**b**) The extraction results obtained under back-lighting conditions; (**c**) The extraction results obtained under cloudy day conditions.

**Figure 14:**
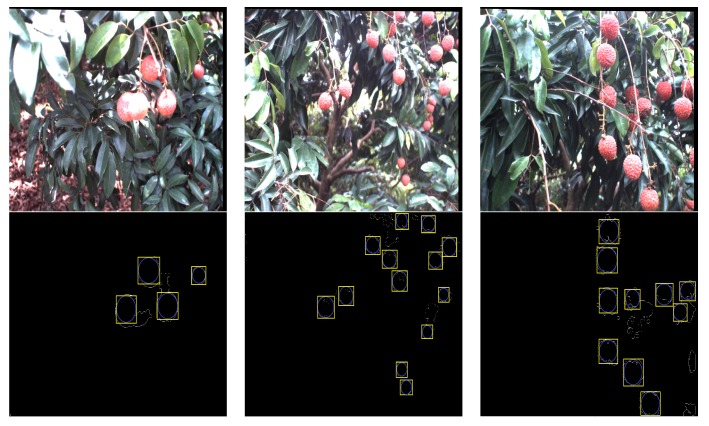
The extraction results of mature litchi fruits including missed clustered litchi fruit. The (**top**) row were the original litchi color images; the (**bottom**) row listed the corresponding extraction results of mature litchi fruits but including missed clustered litchi fruit.

In order to illustrate the performance of the proposed recognition algorithm of clustered litchi fruit under natural environmental conditions, litchi color images were captured under sunny front-lighting conditions, sunny back-lighting conditions and cloudy day conditions, which were shown in Figure 11a–c, respectively. Using the four different classifiers to extract mature litchi fruits of the original litchi color images in Figure 11, respectively, the results were seen in Figure 12, which indicated the results were not ideal based on the single classifier. Under sunny front-lighting conditions, there were some holes on the surfaces of the recognized litchi fruits, as seen in Figure 12a,d,g,j. Although there were fewer holes on the surfaces of the recognized litchi fruits under sunny back-lighting conditions and cloudy day conditions, some recognized fruits were divided into several small irregular portions due to the occlusion of branches or leaves, which were feature points that were difficult to be searched or implemented Figure 12b,c,e,f,h,i,k,l. However, the extraction results of the mature litchi fruits based on combining the results of the four different classifiers were satisfied, and the regular litchi label could completely represent the clustered litchi fruits, which can be seen in Figure 13. Figure 13a shows the extraction result of the mature litchi fruits in the litchi color image obtained under sunny front-lighting conditions. Figure 13b was the result obtained under sunny back-lighting conditions, and Figure 13c was the result obtained under cloudy day conditions. The top row in Figure 14 showed the original litchi color images and the bottom row in Figure 14 showed the results obtained by using our proposed method, where some clustered litchi fruits were missed. We defined the missed clustered litchi fruits as follows:

- Missed single litchi fruit: If the recognized fruit was less than 25% of the actual fruit in the category of single litchi fruit, the single litchi fruit would be considered as the missed single litchi fruit;
- Missed two clustered litchi fruits: If there were one or two recognized fruits less than 25% of the actual fruits in the category of two clustered litchi fruits, the two clustered litchi fruits would be considered as the missed two clustered litchi fruits;
- Missed multiple clustered litchi fruits: If there were two or more recognized fruits less than 25% of the actual fruits in the category of multiple clustered litchi fruits, the multiple clustered litchi fruits would be considered as the missed multiple clustered litchi fruits.

Therefore, true positive rates, false positive rates, false negative rates, precision, recall and F1 of the proposed method under three different illumination and two kinds of occlusion conditions are shown in Table 1. The true positives rate, which was the rate of correct fruit recognition, was 92.82% under six different conditions. The false positives rate, which was the rate of incorrect identifying non-fruits into fruits, was 10.89% under six different conditions. The false negatives rate, which was the rate of incorrect identifying fruits into non-fruits, was 7.18% under six different conditions. The precision, recall and F1 were 89.11%, 92.82% and 90.93% under six different conditions, respectively. The highest true positives rate was 94.17%, achieved under sunny back-lighting and partial occlusion conditions. The lowest true positives rate was 91.07% under sunny front-lighting and partial occlusion conditions. The true positives rates were 92.00%, 92.11%, 93.58% and 93.33% under other conditions. The proposed algorithm received the lowest false positive rate, 6.67%, under cloudy day and non-occlusion conditions. However, the highest false positive rate was 28.13%, happened under sunny front-lighting and non-occlusion conditions. The false positive rates were 10.53%, 9.49%, 12.50% and 9.73% under other conditions. The lowest false negatives rate was 5.83% under sunny back-lighting and partial occlusion conditions. The highest false negatives rate was 8.93% under sunny front-lighting and partial occlusion conditions. The false negatives rates were 8.00%, 7.89%, 6.42% and 6.67% under other conditions. The highest precision, recall and F1 were 93.33%, 94.17% and 93.33% under cloudy day and non-occlusion, sunny back-lighting and partial occlusion and cloudy day and non-occlusion conditions, respectively. The lowest precision, recall and F1 were 71.86%, 91.07% and 80.69% under sunny front-lighting and non-occlusion, sunny front-lighting and partial occlusion and sunny front-lighting and non-occlusion conditions, respectively. The precision, recall and F1 were 89.47%, 91.51%, 87.50% and 90.27%, 92.00%, 92.11%, 93.58% and 93.33% and 90.26%, 92.82%, 89.75% and 91.90% under other conditions, respectively.

### 3.2. Performance of Clustered Litchi Fruit Matching Algorithm

**Figure 15:**
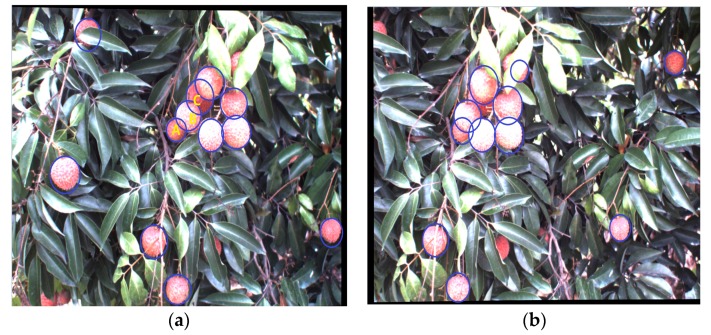
The matching result of the single litchi fruit using CHT. (**a**) Litchi left image; (**b**) Litchi right image.

The matching result of single litchi fruit based on CHT seen from Figure 15 showed some mistaken matching. There were no matching litchi fruits in the right image for litchi A, B and C. The reason was that litchi A, B and C seriously partially occluded each other in the left image, and there were no similar radii and coordinates of the centers with litchi fruits in the right image after circle fitting was implementing on the same rows. It was seen that the single litchi fruit matching easily caused matching errors due to the partial occlusion under natural litchi growth conditions. After the litchi matching experiment, the matching success rates of the litchi fruit under different illumination and occlusion conditions using CHT and the proposed method were recorded in Table 2. Here, 80.04% of the total numbers of the single litchi fruits were successfully matched under six different conditions by using CHT. The highest matching success rate was 82.96% under sunny back-lighting and non-occlusion conditions. The lowest matching success rate was 71.97% under cloudy day and non-occlusion conditions. The matching success rates were 79.08%, 80.68%, 81.39% and 81.89% under other conditions. 

By comparison, 93.75% of the total numbers of the clustered litchi fruits were successfully matched under six different conditions by using the proposed method. The highest matching success rate was 97.37% under sunny back-lighting and non-occlusion conditions. The lowest matching success rate was 91.96% under sunny front-lighting and partial occlusion conditions. The matching success rates were 96.00%, 95.15%, 92.66% and 93.33% under other conditions. 

### 3.3. Real Time Performance of the Proposed Algorithm

The time consumed by the proposed algorithm mainly occurred during clustered litchi fruit extraction and clustered litchi fruit matching based on the geometric centers of the fruit label. The time consumed by the extraction of clustered litchi fruits mainly occurred during searching the fruit regions based on the four different classifiers, and the total average time of processing which achieved 1092 ms. Moreover, traversing the label template of the clustered litchi fruit of the left image into the right image to search for the optimal matching window based on the normalized cross-correlation similarity measure function cost time; the average processing time for a pair of images was 1364 ms. However, the proposed method of clustered litchi fruit label extraction operating on the binary image consumed few time. The average time consumed from the extraction of clustered litchi fruit to fruit localization was 2536 ms.

## 4. Discussion

To recognize and match mature litchi fruit under natural environment, the fruits firstly were categorized into three categories of clustered fruits. Then, four different classifiers were used to extract mature litchi fruits from the litchi color images, respectively. By combining the results, a pixel threshold method was proposed for the recognition of the clustered litchi fruit, which could take advantage of the four classifiers and weaken the effect of varying illumination and occlusion on fruit recognition. The litchi fruits in the orchard under different lighting conditions were tested by combining the recognition results of the four different classifiers and the label extraction method of the clustered litchi fruit. The experimental results demonstrated that the method could well identify the clustered litchi fruit from background (e.g., leaves, branches and sky). The performance tests of the fruit recognition method indicated that the method could partly account for the robustness against the influence of the varying illumination and occlusion. Based on the successful fruit recognition, a clustered litchi fruit matching method based on a geometric center of the cluster label was proposed, which could not only utilize the regular graph advantages of the label in matching, but also avoid mistaken matching disadvantages of the single litchi under occlusion conditions. The experimental results implied that the proposed matching method could obtain more accurate matching result than the result of single litchi matching obtained by using CHT. From the interactive performance of the developed approach in the tests, the average processing time from the extraction of clustered litchi fruit-to-fruit matching was 2536 ms, so it could meet the requirements of harvesting robots [36]. This demonstrated performance indicates that the approach could successfully and robustly recognize and locate mature litchi fruits in the orchard, and it could be embedded into the vision system of a litchi harvesting robot to help it grasp litchi fruits accurately.

However, there were still some shortcomings of the developed method. First, when the illumination intensity in the orchard changes dramatically, the color and texture of the acquired litchi color images will inevitably change. Although the training samples of the constructed classifier have included three kinds of different illumination and six primary textures, it could not cover all illumination conditions, so some parts of the pixels may be wrongly classified and recognized. Second, when most of the clustered litchi fruits was occluded, some clustered litchi fruits may not be mistakenly matched. Therefore, further research is needed to improve the matching accuracy of occluded clustered litchi fruit in the orchard. 

Based on the discussions above, it can be seen that the proposed method can be used for a litchi harvesting robot working in the orchard under natural environment. However, additional research is still needed to improve the recognition and matching rates for satisfying more varied natural environments.

## 5. Conclusions and Future Work

To comply with the development of fruit harvesting robots, an approach to recognize and match clustered mature litchi fruits under natural environmental conditions was developed in this paper. At the beginning of the approach, mature litchi fruits were categorized into three different categories of clustered litchi fruits based on the distance relationship between fruits. The effective color components of R-B, I, Cb and b*, and six primary texture features, which could well separate the mature litchi fruits from background were extracted, based on which the four different classifiers were built, respectively. To identify the clustered litchi fruits, the result of single litchi recognition using each classifier was converted into a binary image, based on which morphology operation and CHT were implemented for label extraction of the single fruit. Then, the four label extraction results based on the four different classifiers were combined for the final single litchi recognition. Lastly, the recognition of the clustered litchi fruit was accomplished by dividing litchi fruits into predefined categories based on the distance between the geometric centers of the single litchi labels. Finally, based on the successful recognition of clustered litchi fruits, the fruit matching was completed using the label of the fruit in the left image to be a template to traverse the right image to search for an optimal window based on the similarity measure method of normalized cross-correlation. Three experiments were implemented by using the litchi images from the real natural environment, and the results were quantitatively assessed and compared with other corresponding methods. The main results are as follows:

(1) The proposed litchi recognition method based on the results of combining the four different classifiers had better recognition results than the results obtained by using single classifier.
(2) The proposed method recognized and matched the clustered litchi fruits instead of single litchi fruits, which made the effects of varying illumination and occlusion on fruit recognition much weaker and improved the recognition and matching accuracy.
(3) The recognition method was able to automatically separate clustered litchi fruits from background, and the accuracy of the classifications could achieve 94.17% under sunny back-lighting and partial occlusion conditions, 91.07% under sunny front-lighting and partial occlusion conditions and 92.82% under natural environment.
(4) The highest and lowest matching success rates of clustered litchi fruits of the proposed method were 97.37% and 91.96% under sunny back-lighting and non-occlusion and sunny front-lighting and partial occlusion conditions, respectively, which were superior to single litchi matching using CHT.
(5) The interactive performance of the proposed algorithm was investigated, and the average consumed time from the extraction of clustered litchi fruit to fruit localization was 2536 ms, which can meet the requirements of litchi harvesting robots.

In conclusion, the developed approach can effectively recognize and match the harvesting targets under a complex natural environment. However, the accurate recognition of the litchi fruits under sever varying illumination conditions and matching of the fruits under fruit occlusion conditions are still issues that need to be solved and will require further research. To solve these tricky problems, in future research we will consider recognition methods of illumination normalization and the matching methods of fruit occlusion.

## Figures and Tables

**Table 1:** The recognition results of the proposed method.

Illumination Conditions	Litchi Clusters	True Positives Rate		False Positives Rate		False Negatives Rate		Precision	Recall	F1
		Amount %		Amount %		Amount	%	%	%	%
SFP	112	102	91.07	12	10.53	10	8.93	89.47	91.07	90.26
SFN	25	23	92.00	9	28.13	2	8.00	71.86	92.00	80.69
SBP	103	97	94.17	9	9.49	6	5.83	91.51	94.17	92.82
SBN	38	35	92.11	5	12.50	3	7.89	87.50	92.11	89.75
CP	109	102	93.58	11	9.73	7	6.42	90.27	93.58	91.90
CN	45	42	93.33	3	6.67	3	6.67	93.33	93.33	93.33
Total	432	401	92.82	49	10.89	31	7.18	89.11	92.82	90.93

SFP = Sunny front-lighting and partial occlusion; SFN = Sunny front-lighting and non-occlusion; SBP = Sunny back-lighting and partial occlusion; SBN = Sunny back-lighting and non-occlusion; CP = Cloudy day and partial occlusion; CN = Cloudy day and non-occlusion; True positives rate = Amount of true positives/(amount of true positives +amount of false negatives) × 100%; False positives rate = Amount of false positives/(amount of false positives + amount of true positives) × 100%; False negatives rate = Amount of false negatives/(amount of false negatives + amount of true positives) × 100%; Precision = Amount of true positives/(amount of false positives + amount of true positives) × 100%; Recall = Amount of true positives/(amount of false negatives + amount of true positives) × 100%; F1 = 2 × Precision × Recall/(Precision + Recall) × 100%.

**Table 2:** The matching results of CHT and proposed method.

Illumination Conditions	Pairs of Litchis	Correct Matching Rate of CHT		Pairs of Litchi Clusters	Correct Matching Rate of Proposed Method	
		Amount	%		Amount	%
SFP	392	310	79.08	112	103	91.96
SFN	88	71	80.68	25	24	96.00
SBP	360	293	81.39	103	98	95.15
SBN	135	112	82.96	38	37	97.37
CP	381	312	81.89	109	101	92.66
CN	157	113	71.97	45	42	93.33
Total	1513	1211	80.04	432	405	93.75

SFP = Sunny front-lighting and partial occlusion; SFN = Sunny front-lighting and non-occlusion; SBP = Sunny back-lighting and partial occlusion; SBN = Sunny back-lighting and non-occlusion; CP = Cloudy day and partial occlusion; CN = Cloudy day and non-occlusion.

## Nomenclature

NIR	near-infrared
RGB	red, green and blue
CCD	charge-coupled device
HSI	hue, saturation and intensity
YIQ	luminance, in-phase and quadrature-phase
YCbCr	luminance, blue chrominance and red chrominance
HSV	hue, saturation and value
BP	Back Propagation
KNN	k-NearestNeighbor
La*b*	luminosity, the range from magenta to green and the range from yellow to blue
SVM	Support Vector Machine
RBF	Radial Basis Function
PC	personal computer
CHT	Circle Hough Transform
NCC	normalized cross-correlation
